# Hesperidin inhibits HeLa cell proliferation through apoptosis mediated by endoplasmic reticulum stress pathways and cell cycle arrest

**DOI:** 10.1186/s12885-015-1706-y

**Published:** 2015-10-12

**Authors:** Yaoxian Wang, Hui Yu, Jin Zhang, Jing Gao, Xin Ge, Ge Lou

**Affiliations:** 1 Department of Gynecology, Third Affiliated Hospital of Harbin Medical University, 150 Hapin Road, Harbin, Heilongjiang Province 150086 China; 2 Cardiopulmonary Function Room, Third Affiliated Hospital of Harbin Medical University, 150 Hapin Road, Harbin, Heilongjiang Province 150086 China; 3 Department of Gynaecology, Fourth Affiliated Hospital of Harbin Medical University, 37 Yiyuan Street, Harbin, Heilongjiang Province 150001 China; 4 Department of General Surgery, Heilongjiang Provincial Hospital, 82 Zhongshan Road, Harbin, Heilongjiang Province 150036 China

**Keywords:** Hesperidin, HeLa cells, ROS, MMP, Apoptosis, ER stress, Cell cycle arrest

## Abstract

**Background:**

Hesperidin (30, 5, 9-dihydroxy-40-methoxy-7-orutinosyl flavanone) is a flavanone that is found mainly in citrus fruits and has been shown to have some anti-neoplastic effects. The aim of the present study was to investigate the effect of hesperidin on apoptosis in human cervical cancer HeLa cells and to identify the mechanism involved.

**Methods:**

Cells were treated with hesperidin (0, 20, 40, 60, 80, and 100 μM) for 24, 48, or 72 h and relative cell viability was assessed using the 3-(4, 5-dimethylthiazol-2-yl)-2, 5-diphenyltetrazolium bromide (MTT) assay.

**Results:**

Hesperidin inhibited the proliferation of HeLa cells in a concentration- and time-dependent manner. Hesperidin-induced apoptosis in HeLa cells was characterized by increased nuclear condensation and DNA fragmentation. Furthermore, increased levels of GADD153/CHOP and GRP78 indicated hesperidin-induced apoptosis in HeLa cells involved a caspase-dependent pathway, presumably downstream of the endoplasmic reticulum stress pathway. Both of these proteins are hallmarks of endoplasmic reticulum stress. Hesperidin also promoted the formation of reactive oxygen species, mobilization of intracellular Ca^2+^, loss of mitochondrial membrane potential (ΔΨm), increased release of cytochrome c and apoptosis-inducing factor from mitochondria, and promoted capase-3 activation. It also arrested HeLa cells in the G0/G1 phase in the cell cycle by downregulating the expression of cyclinD1, cyclinE1, and cyclin-dependent kinase 2 at the protein level. The effect of hesperidin was also verified on the human colon cancer cell HT-29 cells.

**Conclusion:**

We concluded that hesperidin inhibited HeLa cell proliferation through apoptosis involving endoplasmic reticulum stress pathways and cell cycle arrest.

## Background

Cervical cancer is the second most common female cancer worldwide, but it is the leading malignancy in incidence and mortality among women in some developing countries [1, 2]. Several treatments are available for cervical cancer, but each of them has obvious drawbacks. Surgical treatment is usually restricted only to patients at early stages of the disease and young patients who are willing to accept the loss of fertility [3]. Radiotherapy and chemotherapy are not specific to cancer cells and often produce severe adverse effects, including gastrointestinal reactions, bone marrow suppression, immune suppression, nerve injury, hair loss, and development of secondary malignancies [3]. Despite advances in treatment, up to 35 % patients will develop recurrent or metastatic disease when the results of initial treatment are poor. New therapeutic strategies must be developed to improve survival. However, finding safer and more efficient treatments remains an arduous task.

Recent studies have focused on the anti-tumor properties of natural products because these medicines may have fewer side effects and may be more suitable for long-term use compared with synthetic medicines. Hesperidin (30, 5, 9-dihydroxy-40-methoxy-7-orutinosyl flavone, HES) belongs to the flavanone class of flavonoids that is found mainly in citrus fruits [4, 5]. It has many pharmacological activities such as antioxidant, anti-inflammatory, and anti-mutagenic effects; inhibition of prostaglandin synthesis; modulation of drug metabolizing enzymes; and inhibition of tumor promoters [6–11]. The effects of HES on the prevention and treatment of disease have recently received considerable attention [12], particularly its anti-neoplastic effects [13, 14]. Dietary HES inhibits carcinogenesis in the urinary bladder, colon, lung, and breast in rat models [15–19]. In addition, in vitro and in vivo studies have demonstrated the potential of HES as a cytotoxic agent against a variety of malignant human cancers such as colon [20–23], pancreatic [24], hepatocellular [25], breast [26], prostate [26] and leukemia [27]. However, the molecular mechanisms for the growth inhibition and cytotoxicity of HES in HeLa cells are poorly understood.

In recent years, apoptosis has emerged as a major mechanism by which anticancer agents eliminate pre-neoplastic or neoplastic cells. It has been shown that HES can induce apoptosis through a number of mechanisms including increasing nuclear condensation and DNA fragmentation [20]. These effects of HES are mediated through regulation of the B-cell lymphoma-2 (Bcl-2) family [24, 28], activation of caspase-9 and -3 [24], induction of cell-cycle arrest [26], elevation of the levels of reactive oxygen species (ROS) [22, 23], and decreased levels of nuclear factor-κB (NF-κB) [25–27]. HES was also found to inhibit cyclooxygenase-2 (COX-2), matrix metalloproteinase-2 (MMP-2), and MMP-9 [29], and regulate phosphorylation of mitogen-activated protein kinases (MAPKs) including c-Jun N-terminal kinases (JNKs) and extracellular signal-regulated kinases (ERKs) [25, 30, 31]. However, there are no available data on the mechanism of HES inhuman cervical cancer HeLa cells in vitro. The aim of the present study was to investigate the potential anticancer effects of HES on human cervical cancer HeLa cells and the underlying molecular mechanisms.

## Methods

### Chemicals and reagents

HES, MTT (3-(4, 5-dimethylthiazol-2-yl)-2, 5-diphenyltetrazolium bromide), and 2, 7-dichlorodihydrofluorescein diacetate (DCFH-DA) were obtained from Sigma Chemical Co. (St. Louis, MO, USA). The primers of cyclinD1 and cyclinE for real-time polymerase chain reaction (PCR) were purchased from Genscript (Nanjing, Jiangsu, China). Antibodies against GADD153/CHOP, GRP78, cytochrome c, apoptosis-inducing factor (AIF), cleaved-caspase-3, cyclinD1, cyclinE1, cyclin-dependent kinase 2 (CDK2), glyceraldehyde-3-phosphate dehydrogenase (GAPDH), and β-actin were purchased from Cell Signaling Technology (Boston, MA, USA). Fluorescence-conjugated secondary antibodies and Dulbecco’s modified Eagle’s medium were purchased from Invitrogen (Carlsbad, CA, USA). All other chemicals were obtained in the highest purity commercially available.

### Cell culture

Human cervical cancer HeLa cells and human colon cancer HT-29 cells were obtained from American Type Culture Collection (ATCC). Cells were cultured in Dulbecco’s modified Eagle’s medium supplemented with 10 % (*v/v*) fetal calf serum, 100 μg/mL streptomycin, and 100U/mL penicillin. Cultures were maintained at 37°C in a humidified incubator in an atmosphere of 5 % CO_2_. Confluent cells were used for the following experiments.

### MTT cell proliferation assay

Cell proliferation was determined by MTT assay. In brief, HeLa cells and HT-29 cells in logarithmic growth phase were seeded into 96-well plates at 1 × 10^4^ cells/well followed by incubation at 37 °C for 24 h to allow attachment. Then the cells were treated with HES (0, 20, 40, 60, 80, and 100 μM) for 24, 48, or 72 h. Six wells were included in each group. MTT (20 μL of 5 mg/mL) was added to each well and incubated at 37 °C for 4 h. The supernatant was discarded and the formazan precipitates were dissolved in 150 μL of dimethyl sulfoxide (DMSO) by gentle shaking for 10 min. After dissolution, absorbance (A) was measured at 490 nm on a microplate reader (Tecan, Meilen, Zurich, Switzerland). Background absorbance of the medium without cells was subtracted from all experimental samples. Percent viability was calculated as [value of drug-treated group (A)/control group (A)] × 100 %. Each assay was carried out three times, and the results were expressed as the mean (± SEM).

### Detection of apoptotic cells by Hoechst 33342 staining and DNA laddering fragmentation

The apoptosis of HeLa cells and HT-29 cells was detected using the Hoechst 33342 assay kit (Beyotime Institute of Biotechnology, China). HeLa cells (2 × 10^5^ cells/well) were seeded into a 6-well plate and treated with HES (0, 40, 80, and 160 μM) for 48 h. Then the attached cells were washed with phosphate buffered saline (PBS) and fixed with freshly prepared 4 % paraformaldehyde for 30 min. After fixation, the cells were washed with PBS and incubated with Hoechst 33342 staining solution for 5 min. After staining, cells were washed with PBS and anti-fade mounting medium (Beyotime Institute of Biotechnology, Haimen, Jiangsu, China) was added, then the cells were viewed with a fluorescence microscope (Nikon Corporation, Tokyo, Japan). Apoptosis, as indicated by condensed and fragmented nuclei, was observed and recorded with the fluorescence microscope.

The HeLa cells (2 × 10^5^ cells/well) were seeded into a 6-well plate and treated with HES (0, 40, 80, and 160 μM) for 48 h. Then the attached cells were washed with PBS and the DNA was isolated from HES-treated and control cells use DNA isolation kit (Beyotime Institute of Biotechnology, Haimen, Jiangsu, China), separated by 1.0 % agarose gel electrophoresis, viewed and photographed by an ultraviolet light gel documentation system.

### Detection of ROS, intracellular Ca^2+^ concentrations and mitochondrial membrane potential (ΔΨm) in HeLa cells by flow cytometry

HeLa cells (1 × 10^6^cells/well) were seeded into a 6-well plate and treated with HES (0, 40, 80, and 160 μM) for 48 h. Then cells were harvested for detection of ROS, intracellular Ca^2+^ concentration, and ΔΨm.

The level of ROS in HeLa cells was examined by flow cytometry (Becton Dickinson Corporation, USA), using DCFH-DA (Sigma). The cells were harvested and washed twice with PBS. The cells were then re-suspended in 500 μL of DCFH-DA (10 μM), incubated at 37 °C for 30 min, and the level of ROS in the HeLa cells was examined by flow fluorescence activated cell sorting (FACS).

The level of intracellular Ca^2+^ in HeLa cells was determined by flow cytometry, using Indo 1/AM (Calbiochem; La Jolla, CA, USA). The cells were harvested and washed twice with PBS. The cells were re-suspended in Indo 1/AM (3 μg/mL), incubated at 37 °C for 30 min, and then analyzed to detect the changes of cytoplasmic Ca^2+^ levels using flow cytometry.

The ΔΨm in HeLa cells was determined by flow cytometry using 3, 3-dihexyloxacarbocyanine iodide (DiOC6) (4 μM). The cells were harvested, washed twice, re-suspended in 500 μL of DiOC6 (4 μM) and incubated at 37 °C for 30 min before being analyzed by flow cytometry to detect the changes in ΔΨm.

To detect ROS, intracellular Ca^2+^ concentration, and ΔΨm in Hela cells pre-treated with BAPTA or NAC, 1 × 10^6^cells/well of HeLa cells were plated into a 6-well plate and pre-treated with BAPTA (a Ca^2+^ chelator, 10 μM) or NAC (a ROS inhibitor, 1 mM) before adding 80 μM of HES for 24 or 4 h incubation. Then levels of ROS, intracellular Ca^2+^concentrations, and ΔΨm were measured using flow cytometry as the same methods described above.

### Cell cycle assay by flow cytometry

The distribution of HeLa cells in the phases of the cell cycle was quantified using flow cytometry. HeLa cells (1 × 10^6^ cells/well) were seeded into a 6-well plate and treated with HES (0, 40, 80, and 160 μM) for 48 h. Then the cells were harvested by treatment with trypsin and centrifuged at 7500 rpm for 5 min. The cells were washed with PBS, and stained with 500 μL of propidium iodide in the dark at room temperature for 15 min according to the manufacturer’s protocol (Beyotime Institute of Biotechnology, Haimen, Jiangsu, China). The cells were then analyzed by flow cytometry. The percentage of cells in the different cell cycle phases (G0/G1, S, and G2/M phase) was calculated using Coulter Epicx XL-MCL DNA analysis software.

### Western blot analysis

Following treatment with HES (0, 40, 80, and 160 μM) for 48 h, HeLa cells were washed with ice-cold PBS and collected in lysis buffer (50 mM Tris, pH 7.4, 150 mM sodium chloride, 1 % NP-40, 0.25 % sodium deoxycholate, 0.1 % sodium dodecyl sulfate (SDS), 1 mM sodium orthovanadate, 1 mM sodium fluoride, 1 mM ethylenediaminetetraacetic acid (EDTA), 1 mM phenylmethanesulfonyl fluoride (PMSF) and 1 μg/mL leupeptin). The supernatant was obtained by centrifugation at 13,500 rpm for 20 min. Total protein was extracted and protein concentration was determined by the Bradford assay.

For Western blots, 120 μg of protein from each sample were subjected to electrophoresis on 12 % SDS-PAGE and separated proteins were transferred onto a polyvinylidene fluoride (PVDF) membrane. The PVDF membrane was blocked with 5 % non-fat milk powder (*w/v*) at room temperature for 2 h, then incubated with the primary antibodies against GADD153/CHOP (1:500), GRP78(1:500), cytochrome c (1:500), AIF (1:500), cleaved-caspase-3 (1:500), cyclinD1 (1:500), cyclinE1 (1:500), CDK2 (1:500), GAPDH (1:1000), and β-actin (1:500) at 4 °C overnight. After washing, the membrane was incubated with fluorescence-conjugated secondary antibodies (anti-rabbit or anti-mouse, 1:10,000) at room temperature for 50 min. GAPDH or β-actin was used as an internal control to account for protein loading and transfer from the gel to the membranes. Bands on the Western blots were quantified using the Odyssey infrared imaging system (LI-COR, USA). All results represent of three independent experiments.

### Statistical analysis

Data were reported as means ± SEM of at least three independent experiments. For statistical analysis, one-way ANOVA was used for comparison of one variance among groups and two-way ANOVA was used for comparison of two independent variances among groups followed by the Tukey’s post hoc test. *P* values less than 0.05 were considered significant.

## Results

### HES-induced morphological changes and anti-proliferation effect in HeLa cells and HT-29 cells

**Fig. 1:**
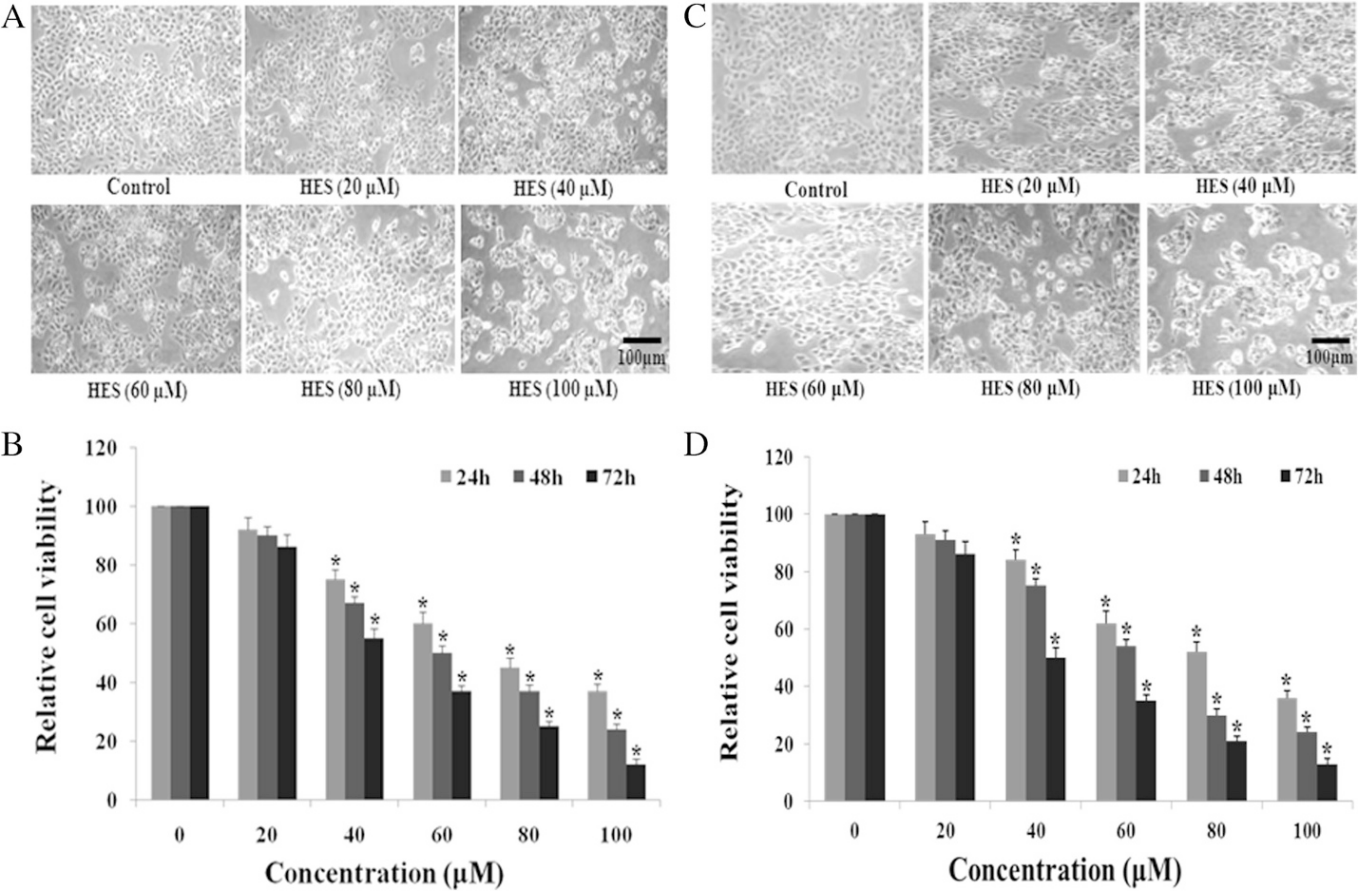
Hesperidin (HES)-induced morphological change and anti-proliferation in HeLa cells and HT-29 cells. **a** and **c** The morphology of the HeLa cells and HT-29 cellswas examined using a phase contrast microscope after treatment with HES. After treatment with HES (0, 20, 40, 60, 80, and 100 μM) for 48 h, Cells showed numerous morphological changes. *Scale bar* = 100 μm. **b** and **d** HES-induced inhibition of proliferation in HeLa cells and HT-29 cells. Cells were treated with HES at concentrations of 0, 20, 40, 60, 80 and 100 μM for 24, 48, and 72 h. MTT assay results are reported as cell viability (%) relative to the control. All data were normalized to the control group, which was set at 100 %. HES inhibited proliferation of HeLa cells and HT-29 cells in a concentration- and time-dependent manner; *p <0.05 versus control group (0 μM) (two-way ANOVA followed by Tukey’s post hoc test)

HeLa cells and HT-29 cells were incubated with HES (0, 20, 40, 60, 80, and 100 μM) for 48 h. The morphology of the cells was examined using a phase contrast microscope. In the presence of HES, HeLa cells showed round morphology with a small amount of shrinkage and nuclear condensation, and a proportion of the cells showed swelling, cell membrane lysis, and disintegration of organelles, suggesting HES-induced toxicity to HeLa cells (Fig. 1a and c).

Cell viability was evaluated by the MTT assay at 24, 48, and 72 h and results were reported as relative cell viability (%). All data were normalized to the control group (100 %). Treatment with HES significantly reduced cell viability compared to the control group (Fig. 1b and d) and the effect of HES on cell viability was concentration-and time-dependent. Cells incubated with 100 μM HES for 72 h showed the maximum anti-proliferative effect, with cell viability decreased to 12 % of the control cells. This result suggests that HES inhibits proliferation of HeLa cells in a concentration- and time-dependent manner.

### HES-induced apoptosis in HeLa cells and HT-29 cells

**Fig. 2:**
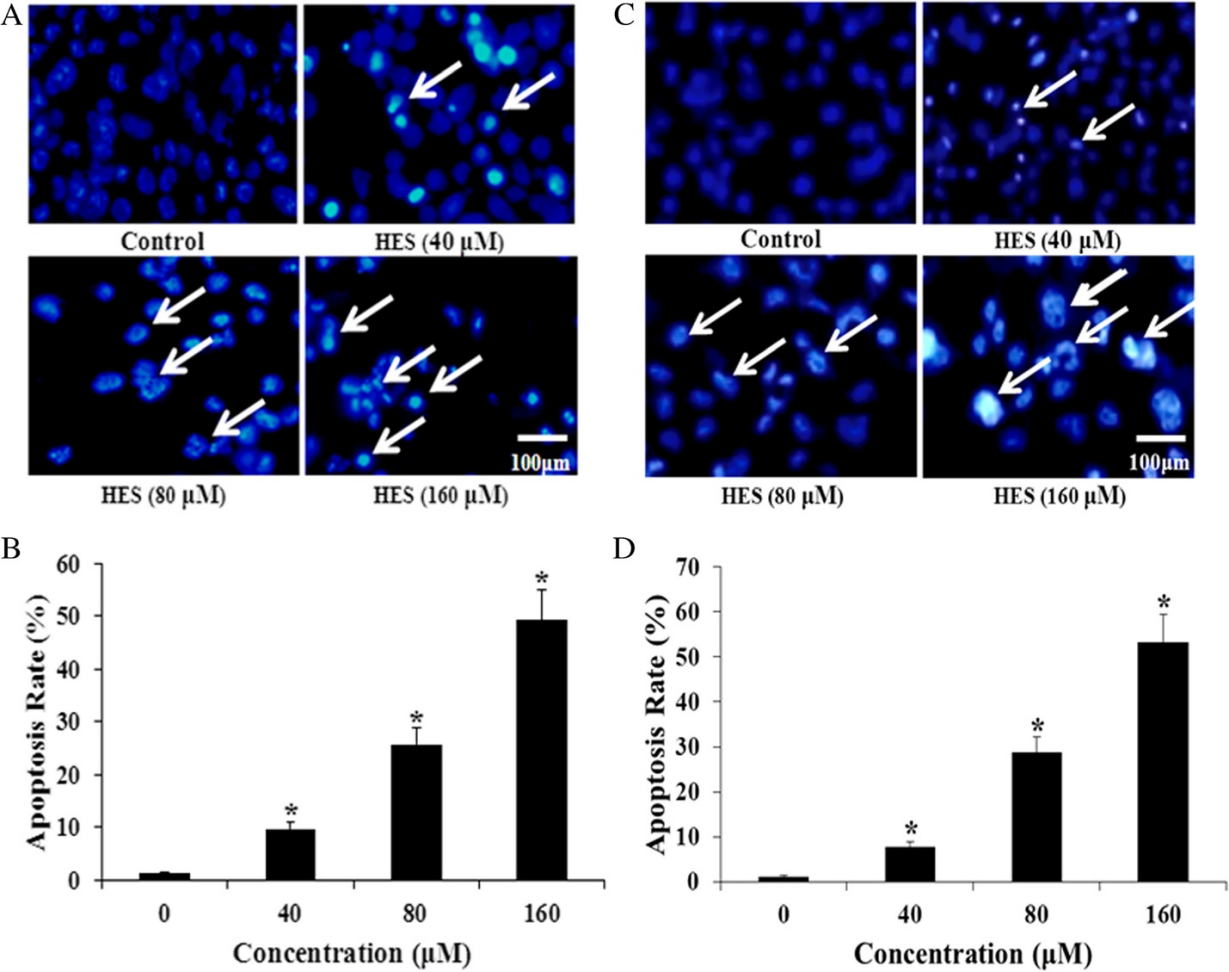
HeLa cell and HT-29 cell apoptosis after treatment with hesperidin (HES) observed using Hoechst 33342 staining. **a** and **c** HeLa cells and HT-29 cells were treated with HES (0, 40, 80, and 160 μM) for 48 h. Apoptotic cells (*Arrows*) exhibited morphological changes in the nuclei typical of apoptosis. *Scale bar* = 100 μm. **b** and **d** HES-induced increase of apoptosis in HeLa cells and HT-29 cells. Cells were treated with HES at concentrations of 0, 40, 80 and 160 μM for 48 h. The level of apoptosis was analyzed by Hoechst 33342 staining; *p <0.05 versus control group (0 μM) (one-way ANOVA followed by Tukey’s post hoc test)

HeLa cells and HT-29 cells were treated with HES (0, 40, 80, and 160 μM) for 48 hand apoptosis was assessed with Hoechst 33342 apoptosis detection kit. Representative images of Hoechst 33342 staining are shown in Fig. 2a and c. HES-treated cells exhibited typical morphological changes indicating apoptosis. The nuclei with condensed chromatin showed more fluorescence than the nuclei in normal cells. Apoptotic HeLa cells also displayed round and shrunken cell bodies (white arrows in Fig. 2a and c). The number of apoptotic HeLa cells increased as the concentration of HES increased (Fig. 2b and d), suggesting that HES-induced apoptosis of HeLa cells might contribute to reduced cell viability.

### HES-induced DNA fragmentation in HeLa cells

**Fig. 3:**
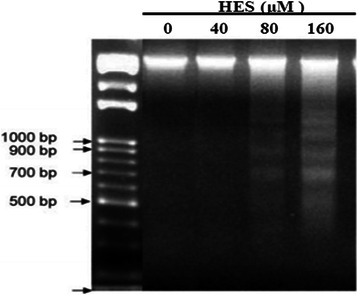
DNA fragmentation as an apoptotic effect of hesperidin (HES) in HeLa cells. HeLa cells were treated with HES (0, 40, 80, and 160 μM) for 48 h and DNA fragmentation was determined using DNA gel electrophoresis

DNA fragmentation is considered another hallmark of apoptosis. HeLa cells were treated with HES (0, 40, 80, and 160 μM) for 48 h and DNA fragmentation was detected using the DNA laddering fragmentation assay. The cleaved DNA fragments in apoptotic HeLa cells were separated by agarose gel electrophoresis (Fig. 3). Staining of the gel with ethidium bromide revealed typical laddering pattern of multimers of 500–1000 bases. Treatment with 80 and 160 μM HES markedly increased DNA fragmentation in HeLa cells. HES induced DNA fragmentation in a concentration-dependent manner.

### HES-induced increase in ROS and cytoplasmic Ca^2+^ levels and decrease in ΔΨm in HeLa cells

**Fig. 4:**
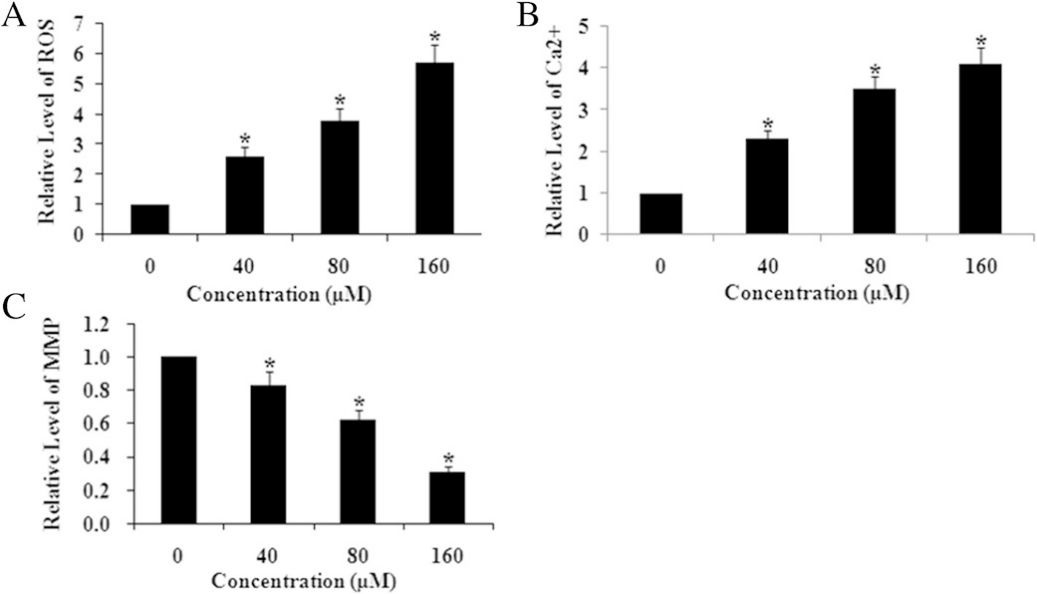
Hesperidin (HES) increased reactive oxygen species (ROS) and cytoplasmic Ca^2+^ and decreased mitochondrial membrane potential (ΔΨm) in HeLa cells. HeLa cells were treated with HES (0, 40, 80, and 160 μM) for 48 h. The levels of ROS (**a**), Ca^2+^ Concentrations (**b**) and ΔΨm (**c**) were analyzed by flow cytometry; *p <0.05 versus control group (0 μM) (one-way ANOVA followed by Tukey’s post hoc test)

To evaluate HES-induced oxidative stress in HeLa cells, the level of ROS was detected by flow cytometry after cells were treated with HES (0, 40, 80, and 160 μM) for 48 h. The level of ROS was increased in the HES-treated groups in a concentration-dependent manner. ROS production was maximal after treatment with 160 μM HES (Fig. 4a).

Intracellular Ca^2+^ homeostasis is critical for normal cell function. Previous studies have suggested that increased cytoplasmic Ca^2+^is associated with mitochondrial dysfunction and leads to overproduction of ROS. To study the impact of HES treatment on Ca^2+^ homeostasis in HeLa cells, cytoplasmic Ca^2+^ was measured by flow cytometry after 48 h treatment with HES (0, 40, 80 and 160 μM). Compared to the control, exposure to 160 μM HES resulted in 3.1-fold increase in cytoplasmic Ca^2+^. The effect of HES on HeLa cytoplasmic Ca^2+^ displayed concentration-dependence over the concentration range tested (Fig. 4b). HES-induced mobilization of cytoplasmic Ca^2+^may be correlated with increased ROS and apoptosis in HeLa cells.

Under normal conditions, mitochondria possess a transmembrane electrical potential (negative inside). A decrease in ΔΨm results in the release of cytochrome c into the intermembrane space that is associated with cell apoptosis. To investigate whether HES affects ΔΨm, HeLa cells were treated with HES (0, 40, 80, and 160 μM) for 48 h and ΔΨm was measured by flow cytometry. Exposure to 40 μM HES decreased ΔΨm by about 20 % and 160 μM HES exposure decreased ΔΨm by up to 70 %. The effectiveness of HES in decreasing ΔΨm exhibited concentration-dependence (Fig. 4c).

**Fig. 5:**
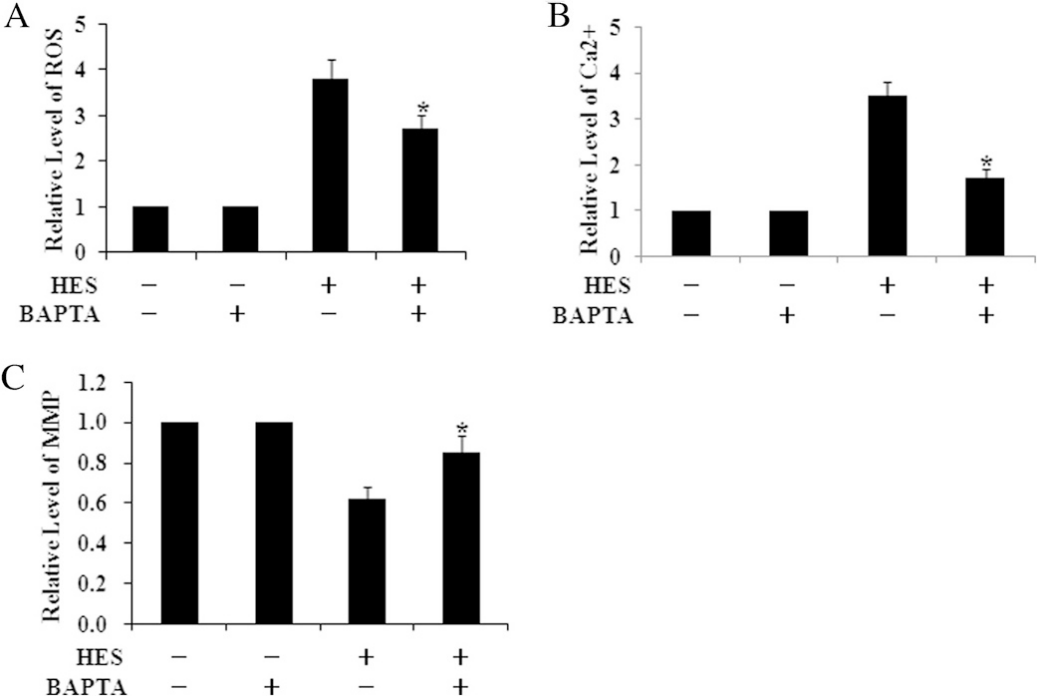
The increase of ROS, Ca^2+^ Concentrations and Mitochondrial Membrane Potential (ΔΨm) level induced by HES (80 μM, 48 h) could be significantly suppressed when pre-treatment with BAPTA for 24 h. The levels of ROS (**a**), Ca^2+^concentrations (**b**) and ΔΨm (**c**) were analyzed by flow cytometry; *p <0.05 versus HES (+) and BAPTA (-) group (One-way ANOVA followed by Tukey’s post hoc test)

**Fig. 6:**
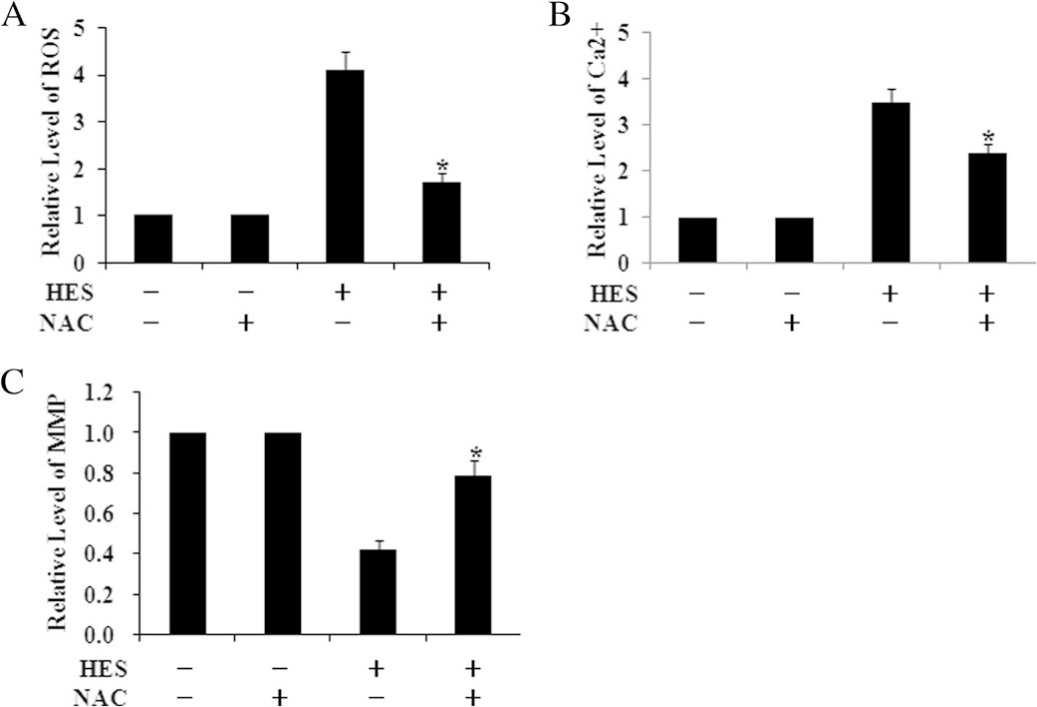
The increase of ROS, Ca^2+^ Concentrations and Mitochondrial Membrane Potential (ΔΨm) level induced by HES (80 μM, 48 h) could be significantly suppressed when pre-treatment with NAC for 4 h. The levels of ROS (**a**), Ca^2+^concentrations (**b**) and ΔΨm (**c**) were analyzed by flow cytometry; *p <0.05 versus HES(+) and NAC(-) group (One-way ANOVA followed by Tukey’s post hoc test)

Above results suggested that HES-induced ROS formation led to cytotoxicity and apoptosis and triggered intracellular Ca^2+^ mobilization. To further test the contribution of calcium mobilization in HES-induced ROS in HeLa cells, we evaluate the effect of NAC, a well-known antioxidant, and BAPTA, a calcium inhibitor, on HES-induced changes of ROS, intracellular Ca^2+^ and ΔΨm in HeLa cells. HES-induced increases of ROS and cytoplasmic Ca^2+^ were significantly suppressed by pre-treatment with 10 μM BAPTA or 1 mM NAC (Figs. 5a-b and 6a-b). Moreover, pre-treatment with BAPTA or NAC inhibited HES-induced decrease of ΔΨm level in HeLa cells (Figs. 5c and 6c). All these results suggested that HES-induced oxidative stress were responsible for HES-induced cytotoxicity and apoptosis through intracellular Ca^2+^ mobilization.

### HES-induced cell cycle arrest at G0/G1 phase of HeLa cells

To gain insight into the mechanism of inhibition of cell growth by HES, effects of HES on the distribution of HeLa cells in the cell cycle was determined by flow cytometry. HeLa cells were treated with HES (0, 40, 80, and 160 μM) for 48 h. HES treatment induced cell cycle arrest in HeLa cells at the G0/G1 phase in a concentration-dependent manner (Table 1). Compared to the control cells (42.10 ± 2.42 % of cells in G0/G1), 40, 80, and 160 μM HES treatment increased the cell population in G0/G1 phase by 1.22-, 1.43-, and 1.71-fold (*p* < 0.05), respectively. HES-induced cell cycle arrest in HeLa cells exhibited a concentration-dependence.

**Table 1:** Hesperidin (HES)-induced cell cycle arrest at G0/G1 phase of HeLa cells

Concentration (μM)	The percentage of cells (%)		
	G0/G1	S	G2/M
0	42.10 ± 2.42	31.70 ± 1.43	26.20 ± 1.31
40	51.70 ± 2.84*	26.95 ± 1.33	21.35 ± 1.06
80	60.30 ± 3.11*	20.45 ± 1.25	19.25 ± 0.97
160	72.20 ± 3.49*	12.10 ± 0.77	15.70 ± 0.82

HeLa cells were treated with HES (0, 40, 80, and 160 μM) for 48 h. The distribution of cells in the cell cycle was measured by flow cytometry; *p <0.05 versus control group (0 μM) (two-way ANOVA followed by the Tukey’s post hoc test)

### HES increased the levels of GADD153/CHOP, GRP78,cytochromec, AIF, and cleaved-caspase-3 in HeLa cells

The above results suggested that HES-induced apoptosis in HeLa cells involved ROS and calcium-mediated mitochondrial pathways in which mitochondrial dysfunction, especially the loss of ΔΨm, led to release of cytochrome c, AIF, and cellular inhibitors of apoptosis (cIAPs) from the mitochondria into the cytosol. These changes resulted in the activation of caspase-3, DNA damage, and cell apoptosis [32–37]. To further elucidate the molecular mechanisms underlying HES-induced apoptosis in HeLa cells, we examined levels of proteins in the endoplasmic reticulum (ER) stress pathway. HeLa cells were treated with HES (0, 40, 80, and 160 μM) for 48 h and protein levels were determined by Western blot.

**Fig. 7:**
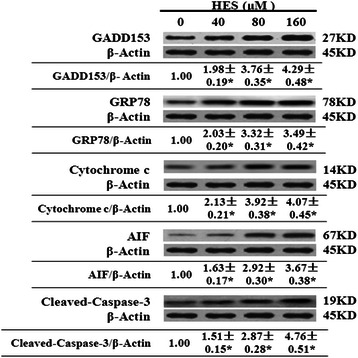
Hesperidin (HES) increased the protein levels of GADD153/CHOP, GRP78, cytochrome c, AIF, and cleaved-caspase-3 in HeLa cells. HeLa cells were treated with HES (0, 40, 80, and 160 μM) for 48 h and the expression of these proteins in treated cells was determined by western blot analysis. Data are reported as the mean ± SEM of at least three experiments; *p <0.05 versus control group (0 μM) (One-way ANOVA followed by the Tukey’s post hoc test)

Exposure to 40 μM HES increased GADD153/CHOP, GRP78, cytochrome c, AIF, and cleaved-caspase-3 protein levels by 1.98-, 2.03-, 2.13-, 1.63-, and 1.53-fold compared to the control (*p* < 0.05), respectively. Exposure to 160 μM HES resulted in the greatest effect on protein levels. The effect of HES on expression of these proteins in HeLa cells was concentration-dependent (Fig. 7).

### HES decreased the levels of cyclin D1, cyclin E1, and CDK2 in HeLa cells

**Fig. 8:**
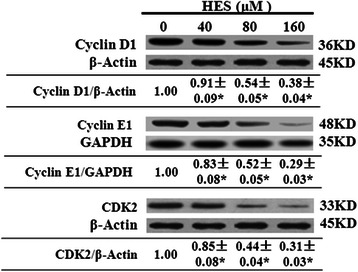
Hesperidin (HES) decreased the protein levels of cyclinD1, cyclinE1, and CDK2 in HeLa cells. HeLa cells were treated with HES (0, 40, 80, and 160 μM) for 48 h and the expression of these proteins in treated cells was determined by western blot analysis. Data are reported as the mean ± SEM of at least three experiments; *p <0.05 versus control group (0 μM) (One-way ANOVA followed by the Tukey’s post hoc test)

We also investigated the effects of HES on markers of cell cycle distribution in HeLa cells by western blot. HeLa cells were treated with HES (0, 40, 80, and 160 μM) for 48 h. In the present study, HES treatment decreased cyclinD1, cyclinE1, and CDK2 protein expression in treated HeLa cells in a concentration-dependent manner. The levels of cyclin D1, cyclin E1, and CDK2 were 0.38-, 0.29-, and 0.31-fold, respectively, of the control level at 160 μM (Fig. 8).

## Discussion and conclusions

HES-induced apoptosis has been previously reported in several cell lines [20–31]. Cell death can be divided into necrosis and apoptosis [38]. Apoptosis is a highly regulated, organized, and programmed death process controlling the development and homeostasis of cells in multicellular organisms [39]. Apoptosis can lead to the death of cancer cells without causing damage to normal cells or surrounding tissues [40]. Thus, induction of apoptosis in cancer cells is a key mechanism for anticancer therapy [41]. In this study, we examined the effect of HES on the human cervical cancer HeLa cell line. We observed a concentration-and time-dependent inhibition of proliferation by HES in these cells. The inhibition effect of hesperidin on human colon cancer HT-29 cells is similar to what previously reported in the literature. We concluded that HES inhibited HeLa cell proliferation through apoptosis through a mitochondria-mediated ER stress pathway and cell cycle arrest.

Our results showed HES induced ROS and intracellular Ca^2+^mobilization. The generation of ROS may contribute to mitochondrial damage and lead to cell death by acting as apoptotic signals [42]. It has been reported that chemotherapeutic agents induce apoptosis in part by generation of ROS and disruption of redox homeostasis [43, 44]. Our data showed increases in intracellular ROS levels by HES as measured by flow cytometry in HeLa cells. ROS production may lead to ER stress and DNA damage.

Cellular Ca^2+^ homeostasis is critical for maintaining normal cell function; depletion of ER Ca^2+^ stores can cause growth arrest and apoptosis [45]. A previous report showed that an increase in intracellular Ca^2+^could induce mitochondrial dysfunction, which in turn resulted in the overproduction of ROS [46, 47], in addition, data from another study indicated that ER stress caused accumulation of ROS leading to cell damage, ATP production decrease, and open of a variety of Ca^2+^ channels in cell membranes and organelles membranes, leading to a significant Ca^2+^ increase in the cytoplasm. Thus a vicious cycle is formed, triggering an apoptosis process in the cells [48]. We used Western Blot to show that HES increased the levels of GADD153/CHOP and GRP78, hallmarks of ER stress. The relationship between ROS or Ca^2+^ and apoptosis has been widely investigated in several cancer cell lines [49, 50].

Mitochondria play an important role in the regulation of apoptosis via mitochondria-dependent and–independent pathways [32, 51]. Characteristics of mitochondrial dysfunction are the loss of ΔΨm, increased permeability, and release of cytochrome c, AIF, and cIAPs from the mitochondria into the cytosol. These effects lead to the activation of caspase-3, DNA damage, and cell apoptosis [33–37, 52]. Our results demonstrated marked activation of caspase-3 by HES presumably resulting from mitochondrial cytochrome c and AIF release into the cytosol followed the decrease in ΔΨm, suggesting that HES-induced apoptosis takes place partly through the mitochondria-mediated ER stress pathway.

It is well known that the mechanism of some chemotherapeutic drugs interferes with the cell cycle [53]. The cell cycle consists of four distinct phases: G1, S, G2, and M. Activation of each phase is dependent on the proper progression and completion of the previous phase. Recent studies suggest that the cell cycle is preferentially arrested in particular phases or phase transitions, such as G0/G1 or G2/M, in drug-induced apoptosis or autophagy-mediated cell death. Therefore, cell cycle distribution may be significant in developing potential chemotherapy drugs.

In response to extracellular signals, cyclin D is an early expression signal and binds with CDK activating the downstream cascade. Cyclin D binds to CDK4, forming anactivecyclinD-CDK4 complex, which then phosphorylates the retinoblastoma susceptibility protein (Rb). Hyper-phosphorylated Rb dissociates from the E2F/DP1/Rb complex activates E2F and results in the transcription of various genes like cyclinE, cyclinA, DNA polymerase, and thymidine kinase. Cyclin E then binds to CDK2, forming the cyclinE-CDK2 complex, which pushes the cells from G1 to S phase [54, 55]. Gene amplification and abnormal expression of cyclinD1 and cyclinE1 have been described in several human cancers [56]. Our results demonstrated that HES resulted in a concentration-dependent increase in the distribution of HeLa cells at the G0/G1 phase and that the process might be mediated through down-regulation of the expression of cyclin D1, cyclinE1, and CDK2.

From these data, we concluded that apoptosis and cell cycle arrest were involved in anti-tumor effect of HES in HeLa cells. HES has been proven to be an effective medicine possessing many pharmacological activities such as antioxidant, anti-inflammatory, and anti-mutagenic effects, inhibition of prostaglandin synthesis, modulation of drug metabolizing enzymes and inhibition of tumor promoters. The anti-tumor effects of HES on cervical cancer in vivo and the underlying molecular mechanisms require further study, but HES has the potential to be developed as a chemotherapeutic or adjuvant agent for human cervical cancer.
